# The Future of Vaccines

**DOI:** 10.3390/vaccines14090832

**Published:** 2026-09-21

**Authors:** Ger T. Rijkers

**Affiliations:** Department of Health, Cognition, and Behavior, University College Roosevelt, 4331CB Middelburg, The Netherlands; g.rijkers@ucr.nl

Humans—as well as all other animals and plant species, for that matter—are surrounded by a world of micro-organisms. Micro-organisms can be grouped into three categories: the good, the bad, and the ugly. Let me explain: the good micro-organisms are the ones that grow on and inside the body—the gut, in particular—and have a health-promoting effect on the host. Early in life, the immune system becomes tolerant of those micro-organisms (also called commensals) and thus a mutual beneficial interaction is established. For the bad micro-organisms, let us use measles as an example. It can cause disease, but in most cases the immune system is able to eliminate the micro-organism. However, not in all cases. When the immune system is not strong enough—for instance, in the case of malnutrition—measles wins. The ugly micro-organisms form a category to which Ebola and tetanus belong. The immune system is not powerful enough to successfully combat these micro-organisms and in the end the micro-organism wins, with the ultimate death of the host (as in most cases of Ebola) [1], or with very severe sequelae (all cases of tetanus) [2]. Of course, there are also many micro-organisms that do neither harm nor good, and in fact have no impact on human health [3].

The immune system can be improved by vaccination so that morbidity and mortality caused by the ugly and bad micro-organisms can be prevented. Vaccines provide the specific activation and induction of immunological memory so that upon future contact with the same micro-organism, the response of the immune system is so fast and powerful that the disease is prevented. Although the function of the immune system can be optimized by a healthy lifestyle, it is not considered a substitute for vaccination.

Outbreaks of diseases caused by ugly and bad micro-organisms in the time before Edward Jenner caused devastating suffering to humans and animals—the most prominent being the plague and smallpox. Jenner was the first person to apply vaccination (with cowpox virus) to prevent smallpox, ultimately resulting in the eradication of the disease worldwide [4]. Further in the past, many outbreaks other than smallpox and the plague may have occurred without us knowing. The current world population are all descendants of survivors of past epidemics and pandemics. The selection of polymorphic genes of the immune system, including but not limited to the major histocompatibility system, has contributed to higher survival rates of subsequent generations [5].

The times after Jenner have seen the rapid development and implementation of safe and effective vaccines against a great number of bacterial and viral diseases, mostly given to children. In many countries, national vaccination programs are in place to ensure that every child, provided that the parents agree, is vaccinated. For low-income countries, international organizations such as the WHO and the Bill and Melinda Gates Foundation offer support [6].

The worth of a vaccine as an effective health intervention depends on its immunogenicity, safety and effectiveness in preventing infections and severe diseases. Vaccine advancements can be realized by enhancing the efficacy and safety of available vaccines as well as by the development of new vaccines with greater efficacy and safety than the available ones. The methods and strategies to generate vaccine advancements can be classified into those associated with the vaccine development; the health outcomes of vaccines in terms of immunogenicity, efficacy, effectiveness and safety; and the access, acceptability and efficiency of vaccination programs.

It is difficult to predict how quickly a safe and effective vaccine against novel diseases can be produced. In the past, Calmette and Guerin worked for 13 years on their vaccine, BCG, for tuberculosis [7]. However, for the COVID-19 pandemic, effective vaccines were developed, tested, and implemented within a year after the outbreak. It is estimated that COVID-19 vaccines in the first year of the pandemic have prevented over 20 million deaths to the SARS-CoV2 virus infection [8]. In the mid 1980s, a novel viral disease emerged: Acquired Immune Deficiency Syndrome, AIDS, caused by the Human Immunodeficiency Virus (HIV). Despite four decades of intense research and many candidates, an effective vaccine is not available yet. A major hurdle could be that there is an incomplete understanding of what constitutes a protective immune response. *Vaccines* aims to publish research aimed at the elucidation of protective immune mechanisms that could lead to novel effective vaccines for all other infectious diseases, both human and animal, for which no vaccines are currently available.

The horrors of vaccine-preventable diseases have faded from the collective memory. Those empty spots now are filled with horror stories in social media of presumed side effects of vaccines. As a consequence, skepticism, hesitancy, and even flat-out refusal of vaccines are on the rise. This has led to the resurgence of some diseases which were a thing of the past, such as measles [9]. Certain religious groups have always rejected vaccination. When members of these groups are evenly mixed within the general population, population immunity protects unvaccinated children and adults. When there are clustered groups within a population which are not vaccinated, either on religious grounds or otherwise, there is an increased risk for outbreaks of vaccine-preventable diseases. What is even more concerning is that vaccines and vaccination have now become a political issue. Political parties, ministries, and complete governments are taking measures that will aid in bringing the vaccination rate down further. Even myths debunked decades ago about the relation between vaccination and autism are returning to government documents [10]. Governments retracting from international organizations slow down the global eradication of diseases like measles and polio. The journal *Vaccines* welcomes and stimulates research on ways to reinstall trust in vaccines among the general population. When this trust is reestablished, policymakers will hopefully follow.

The future of *Vaccines* will see vaccines not only against infectious diseases, but also cancer and autoimmune diseases. A good example of an anti-cancer vaccine is the vaccine against Human Papillomavirus (HPV) [11]. Climate change will have an impact on the spread of infectious diseases. Thus-far-neglected diseases such as river blindness and sleeping disease may spread to areas where climates still remain temperate. This should spur more active research and vaccine development in these areas. A “que sera sera” attitude will not be sufficient; active policies in this regard will be needed.

As with all other scientific journals, *Vaccines* faces the challenge of generative AI. Not just the text, but the research question, experimental design, the data—everything can be artificially generated. This also applies to reviewer’s reports [12]. For a period, the use of AI can certainly help someone’s career, until it is discovered that they are using it. However, the bigger problem is how this affects everyone else. Other scientists end up building their own work on top of results that might be incorrect or flawed. At *Vaccines*, we take this seriously. We do our best to catch these issues during review, and we act on them when we find them.

At *Vaccines*, we are proud to welcome and present the research and perspectives of many across the world who are working on the topics described above, and on other important topics related to vaccines and vaccination. With an open access policy, we strive to contribute to furthering the improvement of the health of all people in the world, especially the most vulnerable among them.

## References

[1] Kayembe B., Matangi B., Matondo P., Okolo A., Mbo P., Tawaba D., Kinuka A., Bepouka B. (2026). Clinical factors associated with mortality in Ebola virus disease: A systematic review and meta-analysis. BMC Infect. Dis..

[2] Chen Z., Lin Z., Zhang W., Hong H., Huang J., Cai Z. (2025). Mortality and risk factors in hospitalized adult patients with tetanus: A systematic review and meta-analysis. BMJ Open.

[3] (2011). Microbiology by numbers. Nat. Rev. Microbiol..

[4] Strassburg M.A. (1982). The global eradication of smallpox. Am. J. Infect. Control.

[5] Klunk J., Vilgalys T.P., Demeure C.E., Cheng X., Shiratori M., Madej J., Beau R., Elli D., Patino M.I., Redfern R. (2022). Evolution of immune genes is associated with the Black Death. Nature.

[6] Shattock A.J., Johnson H.C., Sim S.Y., Carter A., Lambach P., Hutubessy R.C.W., Thompson K.M., Badizadegan K., Lambert B., Ferrari M.J. (2024). Contribution of vaccination to improved survival and health: Modelling 50 years of the Expanded Programme on Immunization. Lancet.

[7] Lobo N., Brooks N.A., Zlotta A.R., Cirillo J.D., Boorjian S., Black P.C., Meeks J.J., Bivalacqua T.J., Gontero P., Steinberg G.D. (2021). 100 years of Bacillus Calmette-Guérin immunotherapy: From cattle to COVID-19. Nat. Rev. Urol..

[8] Watson O.J., Barnsley G., Toor J., Hogan A.B., Winskill P., Ghani A.C. (2022). Global impact of the first year of COVID-19 vaccination: A mathematical modelling study. Lancet Infect. Dis..

[9] World Health Organization Measles Reported Cases and Incidence. https://immunizationdata.who.int/global/wiise-detail-page/measles-reported-cases-and-incidence.

[10] Eicher T., Quackenbush J., Ne’eman A. (2026). Challenging Claims of an Autism Epidemic—Misconceptions and a Path Forward. N. Engl. J. Med..

[11] Henschke N., Bergman H., Buckley B.S., Crosbie E.J., Dwan K., Golder S.P., Kyrgiou M., Loke Y.K., McIntosh H.M., Probyn K. (2025). Effects of human papillomavirus (HPV) vaccination programmes on community rates of HPV-related disease and harms from vaccination. Cochrane Database Syst. Rev..

[12] (2026). Peer review in the time of artificial intelligence. Nat. Nanotechnol..

